# Acquisition and analysis of GFAAS data

**DOI:** 10.1155/S1463924688000240

**Published:** 1988

**Authors:** M. J. Adams, G. J. Ewen, C. A. Shand

**Affiliations:** 1 The Macaulay Land Use Research Institute Craigiebuckler Aberdeen AB9 2QJ UK; 2 School of Applied Sciences The Polytechnic Wulfruma Street Wolverhampton WV1 1SB UK

## Abstract

Since its inception as an analytical technique some 30 years ago atomic absorption spectrometry has become a firmly established method for the analysis of trace metals. Graphite furnace atomic absorption spectrometry provides the analyst with the capability of analysis of solutions containing μg l^-1^ levels of the analyte, but, because of the transient nature of the signals, a sophisticated approach to the data aquisition and handling of data is required. Most modern commercial graphite furnace atomic absorption spectrometers have built in microprocessors for this purpose but they often have limited capability for extensible user programs and limited data storage facilities. In this communication we describe the use of an Apple IIe microcomputer for the acquisition of data from a Pye Unicam SP9 graphite furnace atomic absorption spectrometer. Details of the interface which utilizes an in-house designed AD converter, and an overview of the Pascal and assembler programs employed are given. The system allows the user to record, store and dump the graphical display of the furnace signalsfor all analyses performed. Files containing details of peak height, and area are formatted on an eight-column spreadsheet. Details of sample type, concentrations of standards, dilutions and replication are entered from the keyboard. The calibration graph is constructed using a moving quadratic fit routine and the concentrations of the analyte in unknown solutions calculated. In addition to this, greater processing power and integration of the data into other analytical schemes can be achieved by exporting the data to other software packages and computers. Details of data transfer between the Apple IIe and an Amstrad PC 1512 are given. Some examples of the use of the system in the development of an analytical methodfor silver in plant material are given.


Journal of Automatic Chemistry, Vol. 10, No. 3 (July–September 1988), pp. 130–134

### Acquisition and analysis of GFAAS data

**M. J. Adams,* G. J. Ewen and C. A. Shand**
*The Macaulay Land Use Research Institute, Craigiebuckler, Aberdeen AB9 2QJ, UK*

*Since its inception as an analytical technique some 30 years ago atomic absorption spectrometry has become a firmly established method for the analysis of trace metals. Graphite furnace atomic absorption spectrometry provides the analyst with the capability of analysis of solutions containing μg $l^{-1}$ levels of the analyte, but, because of the transient nature of the signals, a sophisticated approach to the data aquisition and handling of data is required. Most modern commercial graphite furnace atomic absorption spectrometers have built in microprocessors for this purpose but they often have limited capability for extensible user programs and limited data storage facilities. In this communication we describe the use of an Apple IIe microcomputer for the acquisition of data from a Pye Unicam SP9 graphite furnace atomic absorption spectrometer. Details of the interface which utilizes an in-house designed AD converter, and an overview of the Pascal and assembler programs employed are given. The system allows the user to record, store and dump the graphical display of the furnace signals for all analyses performed. Files containing details of peak height, and area are formatted on an eight-column spreadsheet. Details of sample type, concentrations of standards, dilutions and replication are entered from the keyboard. The calibration graph is constructed using a moving quadratic fit routine and the concentrations of the analyte in unknown solutions calculated. In addition to this, greater processing power and integration of the data into other analytical schemes can be achieved by exporting the data to other software packages and computers. Details of data transfer between the Apple IIe and an Amstrad PC 1512 are given. Some examples of the use of the system in the development of an analytical method for silver in plant material are given.*

### Introduction

Atomic absorption spectrometry (AAS) is an established analytical technique, which is routinely employed in many analytical laboratories for the determination of trace quantities of metals in solutions [1]. Since its development by Walsh over 30 years ago the growth in the application of AAS has become part of the trend towards increasing use of instrumentation in chemical analysis. The first commercial AAS instruments were introduced in the early 1960s and generally employed flames as cells for the production of analyte atoms. The flame is still the most common atom cell for routine analyses and, in general, analytical sensitivities range from μg $l^{-1}$ to mg $l^{-1}$. If greater sensitivity is required, or the sample size is inadequate for nebulization into a conventional flame system, then non-flame techniques can be employed. Of these, the most widely used is graphite furnace atomic absorption spectrometry (GFAAS). An increase in sensitivity of between one and three orders of magnitude can be realized for most elements using GFAAS compared with flame AAS procedures. GFAAS has some disadvantages compared with flame AAS. The duration of the analytical signal from a flame system can be extended to any convenient period, provided there is sufficient sample, and the signal can be integrated over this time to achieve a high measurement precision. The signal produced by electrothermal excitation in GFAAS, however, is transitory and requires a more sophisticated approach to the techniques of data capture and recording. Continued improvements and refinements of instrumentation, in particular the application of microelectronic technology, have removed or reduced many of the problems associated with early GFAAS techniques. Most modern commercial instruments used for GFAAS have some built-in microprocessor-controlled unit for recording the analytical signal and many have sophisticated graphic display output facilities and video screen to show the signal trace, the shape of which can be a useful aid in optimizing and monitoring the analytical procedure.

The built-in microprocessors associated with most commercial analytical instruments are programmed to attend to routine tasks and have only limited capability for extensible user programs and, usually, they provide minimal data storage facilities. Where greater data processing facilities are required and there is a need to store data and provide an integrated data management scheme within a laboratory environment, then interfacing the instrument to a personal microcomputer can present the user with a more flexible and extensible analytical system. A wide variety of inexpensive personal computers are available and most are capable of being interfaced to analytical instrumentation. One such machine, which has found extensive application in many laboratories, is the 8-bit Apple II microcomputer.

In this communication we describe the use of an Apple IIe microcomputer for the acquisition and recording of analytical data directly from a Pye Unicam SP9 Video Furnace AAS system (Pye Unicam Ltd, Cambridge, UK). The details of the computer-spectrometer interface are provided and the use of the system is illustrated with typical results obtained from an optimization study of the determination of silver in plant material. An overview of the Pascal and assembler programs employed in the data logging and manipulation procedures is provided. The transfer of data acquired by the Apple IIe from the spectrometer to a second microcomputer operating a commercial spreadsheet and graphics programs is also described.

Present address: Dr M. J. Adams, School of Applied Sciences, The Polytechnic, Wulfruma Street, Wolverhampton WV1 1SB, UK.

© 1988 The Macaulay Land Use Research Institute.

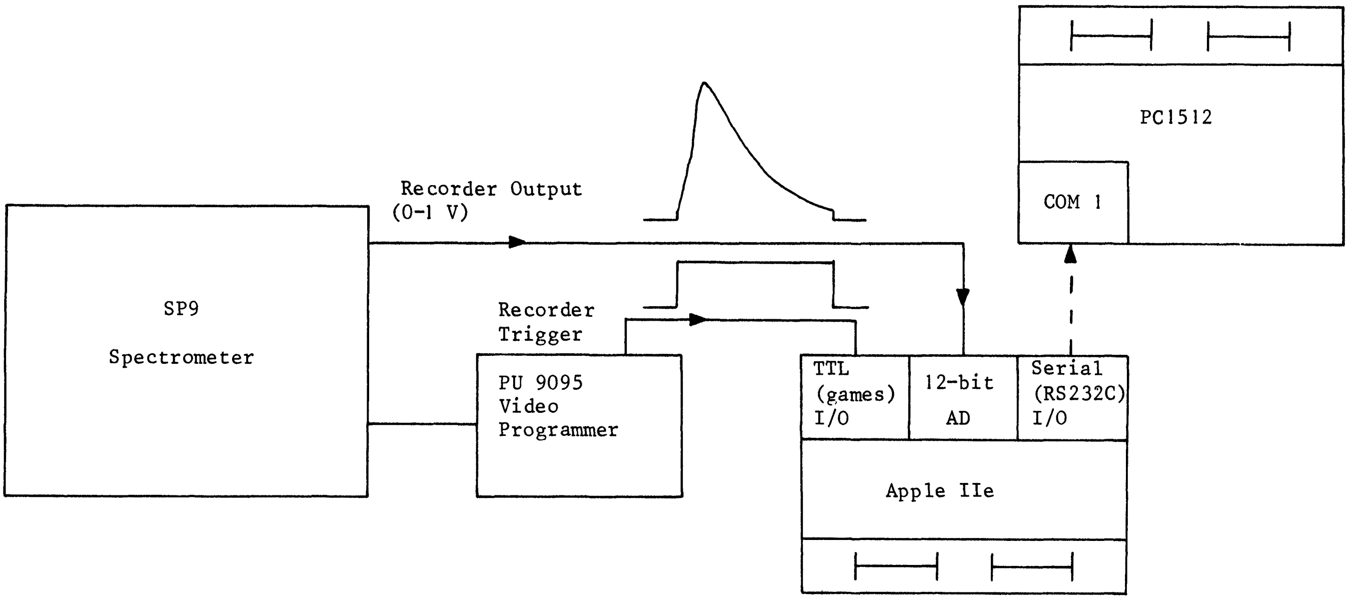

*Figure 1. The general components of the computerized GFAAS system and the interfacing links.*

### Instrumentation

The commercial atomic absorption spectrometer employed is a Pye Unicam SP9 system complete with a PU9095 video furnace programmer, a PU9090 data graphics system and SP9 furnace autosampler. The SP9 is a single-beam spectrometer in which background correction for non-specific absorption is achieved with the aid of a deuterium-filled hollow cathode continuum source which is pulsed alternately with the analyte's hollow cathode lamp. The intensity of the analytical signal output from the photomultiplier is converted to absorbance using a logarithmic amplifier and this signal is output to the data graphics system. In conjunction with the video furnace programmer this data processing system can display 'cookbook' details of analytical conditions for the elements determinable by AAS. In addition, the graphics facilities of the video programmer permit the display of the transient peaks observed in GFAAS studies and the calibration plots for the analytical standards employed. Autosamplers are a desirable addition to any GFAAS system both to improve measurement precision and enable automation of the analytical procedure to be achieved. The SP9 furnace autosampler employed here accepts up to 38 sample cups and two wash cups.

The microcomputer employed to interface to the SP9 system was an Apple IIe. This microcomputer has proved to be a popular choice for laboratory applications largely because of the ease with which interface and expansion circuits can be accommodated within the microcomputer. The Apple IIe machine uses a 8-bit microprocessor (type 6502) as the central processing unit and can directly access up to 64K of memory, which can be expanded to 128K using page-switching techniques. As standard, the computer was equipped with dual floppy disk drive units, a video monitor and an Epson FX-80 printer. Interface circuit cards were installed in the computer using its backplane bus structure; several edge connector slots are available for these boards. All the computer software was written under the Pascal UCSD Operating System.

### Interfacing

The general interfacing scheme is illustrated in figure 1. The spectrometer was connected to the microcomputer via an analogue-to-digital (AD) converter within the computer and its so-called games socket which served as a TTL trigger detector. The AD converter was designed and constructed in-house and has been described in detail elsewhere [2 and 3]. It comprises a precision 12-bit CMOS AD574 integrated circuit (Analog Devices) which can complete a 12-bit data conversion in about 25 μs. This device and all other components, including the X10 signal amplifier, were mounted on a single circuit board for direct connection to the computer using one of its input/output expansion slots. The AD converter was operated in a unipolar mode, 0–10V FSD. The analogue analytical signal was taken directly from the spectrometer. Normally, this signal is supplied as a voltage in the range 0–10 mV, corresponding to 0–1 Absorbance Units, for use with a standard analogue chart recorder. To eliminate potential problems due to electrical pick-up on the spectrometer-computer cable the absorbance and scale expansion circuit board within the spectrometer was modified to provide a 0–1V analogue output. This was achieved by bypassing an internal signal attenuator circuit.

In employing analogue signals for digital data acquisition programs, special care must be taken to control the initiation of the data logging procedure and the rate at which data is accumulated. In the application discussed here the cycle start pulse was obtained from the recorder socket of the video furnace programmer. This signal is conventionally used to activate a high-speed chart recorder, which can be employed to monitor the transient analytical trace. This signal (0/5V TTL) was led to the games socket of the Apple computer. The rate of data acquisition was under program control and selected to achieve about 450 readings in the maximum 9s analytical measurement period.

The microcomputer interfacing and data acquisition software was written in Pascal with the AD conversion routine being prepared as assembly code and called from the main program as an external function. Once initiated, this program strobes the computer keyboard and the TTL (games) socket to test if a quit key has been pressed by the operator or if an analytical program has been started from the video programmer. If an analytical cycle is initiated then digitization and recording begins. The spectrometer system is programmed to provide a 7s autozeroing period prior to firing the graphite furnace and during this period 350 digital readings are recorded and averaged by the Apple computer to provide a mean background reading which is subtracted from each point of the resultant analytical AAS trace. The AAS data is recorded until the trigger signal is deactivated as programmed at the video furnace programmer. Following each analytical cycle the Apple computer scales the AAS data and presents the complete trace on its graphics display unit along with the maximum signal recorded (peak absorbance) and the signal area (absorbances). The trace data is stored in a disk file after each analysis to minimize data loss should there be a program crash or any loss of electrical power to the computing system. The whole procedure is repeated until the quit key is pressed by the user. When the data acquisition program is exited the analytical data in the disk file is re-formatted to provide a data file containing the peak height and area data and a graphics file containing the original data for the profile of the trace. These files can be used for subsequent calibration and manipulation programs within the Apple computer or exported to other computer systems. A second computer, an Amstrad PC1512, was employed for off-line processing and management of the analytical data. The Apple microcomputer was connected to the PC via a serial, RS232C, interface link. Data transferred to the PC could be studied using a wide range of commercial software packages and integrated with other analytical and management data in this second microcomputer.

### Data processing and analysis

The principal data processing software for manipulating and analysing the pre-recorded GFAAS data is an in-house program, DataCalc. DataCalc loads the analytical data into computer memory and displays it a screen 'page' at a time, on the video monitor. The format of the display is similar to many of the common spreadsheet programs, viz. the analytical data occupies discrete rows and columns in the screen. The user can move a cursor from cell to cell between columns in a record and between records or rows. The data can be edited, listed on the printer and saved back on floppy disk for storage and future processing operations. Data processing commands include the determination of the analytical calibration curve and fitting the sample absorbance data to this curve. The command structure of DataCalc is illustrated in figure 2.

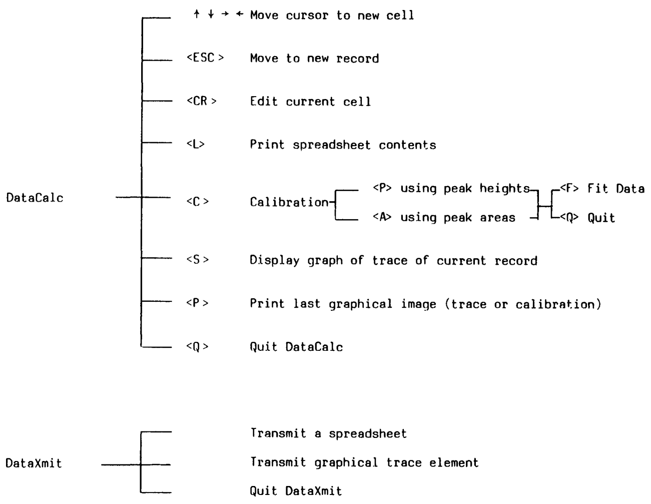

*Figure 2. The command structure of the Pascal DataCalc spreadsheet program used to analyse the GFAAS data.*

As well as the date and filename, displayed on the top row of the screen, up to 16 records (rows) of analytical data, formatted in eight columns are displayed at any time. These data columns provide the record (sample) number, class, replicate code, dilution factor, analytical signal peak height and area and the computed, or user-entered, concentrations. The record class defines the nature of the sample as being a standard, unknown, blank or null. Any record marked by the user as being null is ignored by the computer in subsequent calculations. Analyses by GFAAS are rarely performed singly. Sample duplication is usually encountered and to indicate this replication, the program permits an integer replicate code to be used to indicate similar solutions, the absorbance or concentration values of which will be automatically averaged. The peak height and area fields contain integer values corresponding to the analytical instrument's signal output, as digitized by the computer (these values can be entered by the user if the program is used in isolation from the spectrometer). The concentration column contains either values for standards keyed-in by the user or values computed following calibration.

The major function of the DataCalc processing package is to compute an analytical calibration curve from the standards' data and calculate previously unknown sample concentrations by interpolating their measured signal absorbance values on the standard curve. The set of standards' records is sorted into an order of increasing concentration, using a simple bubble-sort routine, and the curve-fitting coefficients are calculated. The procedure adopted to compute the calibration graph is similar to that employed by the spectrometer manufacturers and detailed by Whiteside *et al.* [4]. The average absorbance value determined from all designated blank solutions is subtracted from all other absorbance data and a straight line is fitted between zero and the first standard. A quadratic function is then fitted between every subsequent pair of data points, a third fitting point being calculated using extrapolated slopes. This 'moving-quad-

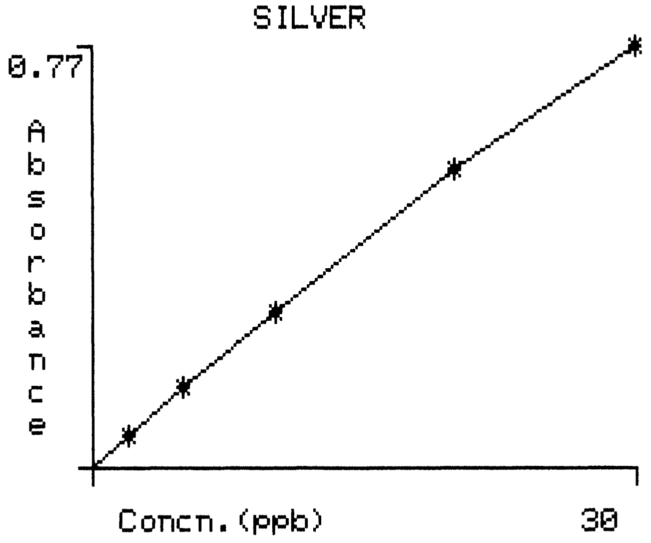

*Figure 3. Screen dump of calibration curve for silver.*

ratic' curve-fitting procedure ensures the curve follows the standards and can be used for complexed shaped calibration graphs. Once calculated, the general form of the calibration curve is displayed on the high-resolution graphics screen of the monitor. If any standard data point is obviously in error, the procedure may be repeated after editing the data by classifying the offending data as a null record. If the curve is accepted then the unknown data points are fitted automatically to the curve and the sample concentration values computed and the results displayed on the screen. The calibration procedure can be undertaken with peak height or area data, as selected by the user.

In addition to these data processing functions the software allows the user to examine the GFAAS profile of any of the records by loading the appropriate data from the disk-based graphics file. The display of this data, or the displays of the analytical calibration curves, can be output to the printer. A printed 'screen-dump' of the calibration curve for the determination of silver by AAS is illustrated in figure 3. The facility to recall and display absorbance profiles on screen allows the operator to quickly examine results from the unattended analysis of a batch of samples to check, for example, that the absorbance profiles of samples and standards are closely similar and that there are no spurious signals. This provides the analyst with a degree of confidence that cannot be obtained from a simple numerical value.

The functions performed by the DataCalc software on the Apple computer are sufficient for most of the routine tasks performed with the GFAAS system. Greater processing power and integration of the AAS data into a more comprehensive analytical scheme can be achieved by exporting the data and results to other software packages and computers. This can be readily achieved by transmitting the data as an ASCII file via a serial RS232C interface from the Apple to a second computer. For most of the time this host machine can be operated independently of the Apple system and will, typically, be running commercial spreadsheet, database, word processing and statistics packages.

A Pascal program can be selected to transmit the analytical data from the Apple machine to the PC system. The user is prompted to select the transfer of absorbance profile graphics, data or the analytical spreadsheet data. Communication is achieved between the two microcomputers using 'Kermit', a *de facto* standard protocol for inter-computer information transfer, operating on the PC. Controlled by the PC the data is output from the Apple and recorded as a text file on a PC disk. This data is available for subsequent analysis.

### Applications

The main function of the computerized system is to collect raw data from the atomic absorption instrument, compute the analytical calibration curve from the standards and calculate the concentration of the analyte in unknown samples. In addition to this routine operation, however, the facilities offered by the microcomputer are valuable during the development of new furnace analytical programs. An example of the use of the system in the development and application of a method for the analysis of silver in nitric acid extracts of ashed sphagnum moss is described.

The first stage in the development of GFAAS methods often involves a series of measurements with standard solutions of the analyte to determine the optimum conditions for the pyrolysis and atomisation steps of the furnace program. Figure 4 shows the profiles for the atomization of 0·1 ng Ag (applied as $AgNO_3$ in 2% $HNO_3$) at 1800 °C from a totally pyrolytic graphite tube and platform combination following pyrolysis at different temperatures.

A comparison of the profile obtained at a pyrolysis temperature of 340 °C with that at 490 °C shows the appearance of an additional early peak. This is indicative of a slight loss of silver from the platform to the tube wall during pyrolysis. This deduction was confirmed by the atomization of Ag from the wall alone, see figure 4(*d*). As the pyrolysis temperature is increased further, to 685 °C, there is in addition to loss from the wall to the platform, a substantial loss of analyte from the atom cell resulting in a decrease in peak height and area. The pyrolysis temperature selected for further work on Ag, applied as nitrate, was 340 °C.

The use of a host computer running a commercial spreadsheet program 'Symphony' is illustrated in figure 5. This shows an overlay of the absorbance profiles for the atomization of 0·1 ng Ag at different temperatures. In this case the data has been re-scaled (DataCalc scales all points to fill the page) using the spreadsheet commands of Symphony. The efect of increasing the atomization temperature on the relative positions and shapes of the signals is clearly illustrated. As the atomization temperature is increased peak height increases sharply and no plateau is reached in the range studied. Peak area measurements exhibit a slight decrease as the temperature is increased, probably due to increased diffusion losses. For subsequent analyses a compromise atomization temperature of 1800 °C held for a period of 5s was

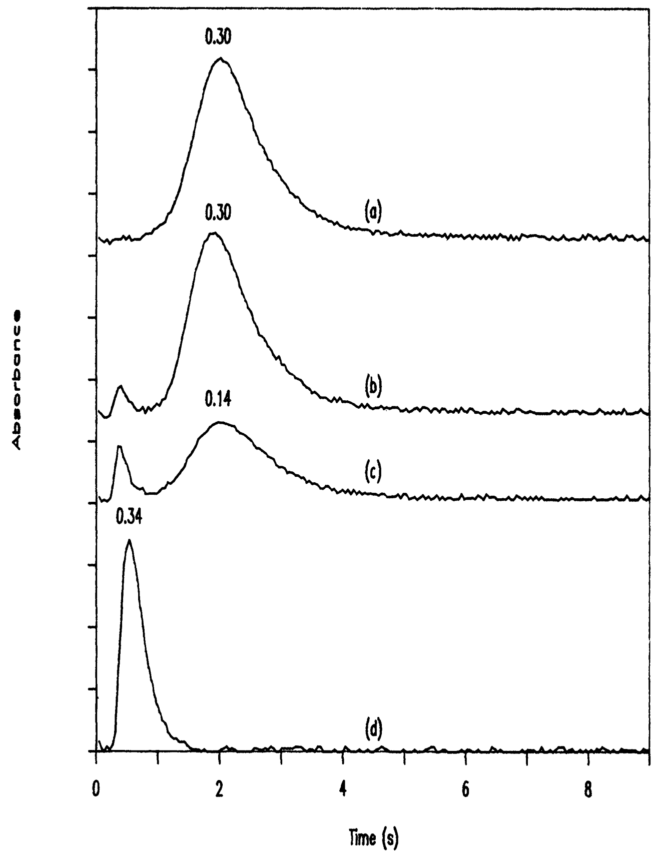

*Figure 4. Absorbance profiles for atomization of 0·1 ng Ag at 1800 °C. (a) following pyrolysis at 340; (b) 490; and (c) 680 °C using a totally pyrolytic tube and platform; and (d) following pyrolysis at 340 °C using a tube alone.*

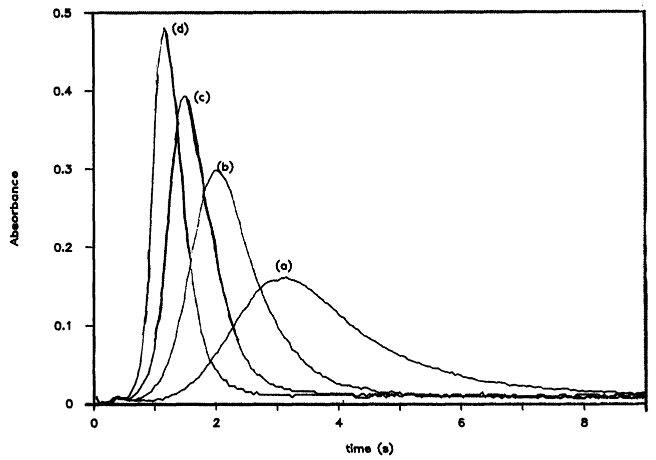

*Figure 5. Overlay of the absorbance profiles for the atomization of 0·1 ng Ag. (a) at 1500; (b) 1800; (c) 2100; and (d) 2400 °C using a totally pyrolytic tube and platform.*

used. This example of the use of a powerful spreadsheet program such as Symphony serves to illustrate the principle of data transfer and use within a laboratory and has many potential applications. Where a detailed study or mathematical treatment of absorbance profiles is to be conducted, for example kinetic modelling of the atomization process, a variety of commercial programs are available. Similarly, the range of data-base packages commercially available for microcomputers makes the in-house development of such programs largely unnecessary. They can form the basis of solphisticated laboratory data management systems which can readily link to the dedicated microcomputer application as detailed here.

### Conclusion

A microcomputer system has been described which interfaces to a commercial electrothermal atomic absorption spectrometer for the direct acquisition and subsequent manipulation of the analytical data. As described above, an important advantage of this computerized system is that a full record can be maintained of the absorbance *versus* time profiles for all the solutions analysed. This information is invaluable in designing new analytical procedures when it is necessary to monitor the effects on the analytical signal of, for example, different ashing and atomizing temperatures. The computerized storage of the graphic trace information also permits the user to check an analysis where a fault is suspected to have occurred in the graphite tube system.

The absorbance peak height and area data are recorded in the computer system for calibration and analysis using spreadsheet-type software. In many cases no further manipulation of this data is required other than that supplied by the in-house designed programs. Where the AAS analysis, however, forms a part of a wider analytical scheme involving other techniques and other laboratories, the GFAAS data recorded by the microcomputer system can be transferred via a simple asynchronous serial interface to other computer systems. An example of GFAAS data manipulation using a commercial spreadsheet package on a PC computer has been presented.

### References

1. Van Loon, J. C., *Selected Methods of Trace Metal Analysis* (John Wiley and Sons, New York, 1985).
2. Ewen, G. J. and Adams, M. J., *Laboratory Practice*, **33** (1984), 116.
3. Adams, M. J. and Ewen, G. J., *Journal of Automatic Chemistry*, **6** (1984), 202.
4. Whiteside, P. J., Stockdale, T. J. and Price, W. J., *Spectrochimica Acta*, **35B** (1980), 795.

